# Disseminated Histoplasmosis in HIV-Infected Patients in South America: A Neglected Killer Continues on Its Rampage

**DOI:** 10.1371/journal.pntd.0002319

**Published:** 2013-11-21

**Authors:** Mathieu Nacher, Antoine Adenis, Sigrid Mc Donald, Margarete Do Socorro Mendonca Gomes, Shanti Singh, Ivina Lopes Lima, Rosilene Malcher Leite, Sandra Hermelijn, Merril Wongsokarijo, Marja Van Eer, Silvia Marques Da Silva, Maurimelia Mesquita Da Costa, Marizette Silva, Maria Calvacante, Terezinha do Menino Jesus Silva Leitao, Beatriz L. Gómez, Angela Restrepo, Angela Tobon, Cristina E. Canteros, Christine Aznar, Denis Blanchet, Vincent Vantilcke, Cyrille Vautrin, Rachida Boukhari, Tom Chiller, Christina Scheel, Angela Ahlquist, Monika Roy, Olivier Lortholary, Bernard Carme, Pierre Couppié, Stephen Vreden

**Affiliations:** 1 Centre d'Investigation Clinique Epidémiologie Clinique Antilles Guyane (Inserm/DGOS CIE 802), Centre Hospitalier de Cayenne, Cayenne, French Guiana, France; 2 Epidemiologie Parasitoses et Mycoses Tropicales, EA 3593, Université Antilles Guyane, Cayenne, French Guiana; 3 Academisch Ziekenhuis Paramaribo Hospital, Paramaribo, Suriname; 4 Laboratório Central de Saúde Pública do Amapá, Macapa, Brazil; 5 National AIDS Program, Georgetown, Guyana; 6 Public Health Central Laboratory of Suriname, Paramaribo, Suriname; 7 Diakonessenhuis Hospital, Paramaribo, Suriname; 8 Instituto Evandro Chagas, Belém, Brazil; 9 Hospital de Clinicas Dr. Alberto Lima, Macapa, Brazil; 10 Universidade Federal do Ceara, Faculdade de Medicina, Departamento de Saude Comunitaria, Fortaleza, Ceara, Brazil; 11 Corporación para Investigaciones Biológicas, Medellín, Colombia; 12 INEI-ANLIS “Dr. Carlos G. Malbrán,” Buenos Aires, Argentina; 13 Centre Hospitalier de l'Ouest Guyanais, Saint Laurent du Maroni, French Guiana, France; 14 Mycotic Diseases Branch, Centers for Disease Control and Prevention, Atlanta, Georgia, United States of America; 15 Institut Pasteur, National Reference Center for Mycoses and Antifungals, Molecular Mycology Unit, Paris, France; 16 CNRS URA3012, Paris, France

HIV/AIDS is not a neglected disease. Histoplasmosis is not considered a neglected disease in North America. However, in South America, it should be. It often affects neglected populations and represents a lethal blind spot of the HIV/AIDS data collection systems. Counts of new AIDS cases and AIDS-related deaths are useful to follow the epidemic; however, they overlook the exact cause of death. In the context of the South American pathogen ecology, the systemic mycosis due to *Histoplasma capsulatum var. capsulatum* is probably on the top of the list of AIDS-defining illnesses and AIDS-related deaths [1], yet it is mostly undiagnosed and is not even on the diagnostic algorithm used by a significant proportion of clinicians facing a febrile, severely immunodepressed patient in the region.

## The Invisible Burden

Studies performed in the 1950s and 1960s on the histoplasmin skin test positivity in South America showed positivity rates around 30% from Trinidad and Tobago in the North, to Uruguay and Argentina in the South. The pathogen is there [2]. Despite this, expertise and awareness of this disease is limited to mycologists and some clinical teams scattered throughout the South American continent [2]–[16]. But those with expertise are the exception rather than the rule. Imported cases in Europe occurring in HIV-infected residents or travellers from South America, notably in France, Spain, and Italy, are starting to be recognized, but often late in the course of the disease because clinicians are not familiar with this “endemic” disease [17]–[19].

For too long, the absence of a simple, reliable, and affordable diagnostic test has made it difficult to determine the burden of this disease in HIV-infected patients in much of South America. The gold standard for diagnosis relies, so far, on the culture of fluid and tissue samples [20]. This requires invasive investigations by clinicians (bone marrow aspirates; biopsies of the liver, lymph node, and intestine; etc.). From the lab perspective, direct examination may accelerate diagnosis, but culture may take weeks and require a BSL 2 laboratory. Detection of specific antigens in serum or urine samples is not available for diagnosis in most of South America, and galactomannan detection (cross reactivity during histoplasmosis) is not being used as a standard of care. Contact with clinicians from various countries suggests that, although severe histoplasmosis often kills in a few days, most clinicians are not very aggressive in their investigations. Moreover, presumptive antifungal therapy is rare. Biopsies are usually immersed in formalin by surgeons rather than sent for culture. Most often, clinicians do not take proper samples for mycological diagnosis, creating a vicious cycle that diminishes the capacity of mycological laboratories and perpetuates underfunding and the absence of diagnosis and, thus, the “nonexistence” of the very disease that is killing numerous patients.

In French Guiana, there has been a mycology laboratory since 1997 with a BSL 3 laboratory. The virtuous cycle between laboratory and clinicians has been fruitful: clinicians are well informed about histoplasmosis and are quite aggressive in looking for the infection, while the mycology laboratory has the capacity to appropriately process and identify *Histoplasma* in clinical specimens [21]. Although published maps [2] show no histoplasmosis in most of French Guiana, the recent figures that have emerged are striking. With 1.5 cases per 100 patient-years, histoplasmosis is the most common cause of AIDS-defining illness. Interestingly, despite awareness of this disease and availability of liposomal amphotericin B, it has also been the leading cause of AIDS-related death for decades. A recent 2-year study of all HIV patients admitted in Saint Laurent du Maroni hospital showed that 41% of admitted patients with CD4 counts below 200 had disseminated histoplasmosis, and 85% of admitted patients with CD4 counts below 50 and isolated fever had disseminated histoplasmosis. This is a clear message for physicians when admitting a severely immunodepressed HIV patient in the region: “*Don't miss histoplasmosis!”*

## No Data = No Existence. Meanwhile, Patients Continue Dying from a Treatable Disease…

The high prevalence of HIV–*Histoplasma* coinfections on the South American continent is not a trivial problem. The scarcity of the published research on this topic reflects the tragic fact that this problem is evolving under the radar of health care systems and is truly a neglected disease. Generations of young doctors will learn to look for tuberculosis, pneumocystosis, and bacterial pneumonia when confronted with a febrile patient with respiratory signs, but not for histoplasmosis. Similarly, important clinical clues, such as cytopenia (ascribed to bone marrow involvement with or without haemophagocytic syndrome) and liver enzyme abnormalities will often not lead to the suspicion of disseminated histoplasmosis. A big danger is that a smear negative, treatment resistant, “tuberculosis-like” syndrome may often be labelled drug-resistant tuberculosis, when it was never tuberculosis in the first place. It is thus of paramount importance to fill this knowledge gap and revise the diagnostic and therapeutic algorithms in the region. The “*Know your epidemic, know your response”* UNAIDS slogan should also be applied to histoplasmosis.

## We Need Research and We Need to Act Now

The HIV/AIDS epidemic is still active in countries in the Amazon basin. Guyana, Suriname, French Guiana, and the Brazilian state of Amapa all have HIV prevalence rates over 1% of the population. Although the AIDS incidence has steadily declined in the southern states of Brazil, the situation in the northern (Amazonas, Roraima, and Amapa) and the northeastern states of Brazil is still concerning. A very coarse calculation based on 600,000 HIV patients and an annual histoplasmosis incidence rate of 1.5% would estimate the annual number of cases to be in the thousands. Unfortunately, histoplasmosis thus still has a future in HIV patients. Although there is a need for epidemiologic research to measure the true burden of disease in various regions of South America, actions can, and should, be taken to diagnose and treat patients now. We need to develop standard mycological practices in the area that emphasize early and aggressive clinical diagnosis, and we need to develop new rapid diagnostic tools and advocate for affordable treatment. New affordable diagnostic assays (CDC, Immy) are presently being tested in Brazil, French Guiana, Suriname, and Colombia. We hope they will allow us to improve our knowledge of local epidemiologies and reduce patient mortality through early diagnosis and increased awareness. Although amphotericin B is available, it has potential significant renal side effects. Liposomal amphotericin B is the treatment of choice of the most severe cases of HIV-associated histoplasmosis, but its cost exceeds 800 US dollars per day. Gilead Sciences, Inc. has committed to the procurement of HIV drugs at affordable prices. Extending this policy to the problem of treating HIV-associated histoplasmosis, a neglected disease, would be an important step in improving the health of HIV/AIDS patients in the region. In the near future, DNDi (Drugs for Neglected Diseases initiative) should provide a low-cost, heat-stable alternative to liposomal amphotericin B that could be valuable for histoplasmosis treatment. Our focus has been HIV-associated disseminated histoplasmosis; however, it should be emphasized that the problem of missing the diagnosis of histoplasmosis in South America also extends to some immunocompetent patients or patients with causes of immunodepression other than HIV [22].

We need tests; we need treatments; but first of all, we physicians need to integrate our South American epidemiology in our diagnostic algorithms. Looking for malaria in febrile patients in malaria-endemic areas is automatic; looking for histoplasmosis in febrile, immunosuppressed, HIV-infected patients in South America, Central America, and perhaps way beyond [23], [24], is not. The sooner we do it, the better for our patients.

## References

[1] Nacher M, Adenis A, Adriouch L, Dufour J, Papot E, et al (2011) What is AIDS in the Amazon and the Guianas? Establishing the burden of disseminated histoplasmosis. Am J Trop Med Hyg 84: 239–240. 21292891 10.4269/ajtmh.2011.10-0251 PMC3029174

[2] Colombo AL, Tobon A, Restrepo A, Queiroz-Telles F, Nucci M (2011) Epidemiology of endemic systemic fungal infections in Latin America. Med Mycol 49: 785–798. 21539506 10.3109/13693786.2011.577821

[3] Arango M, Castaneda E, Agudelo CI, De Bedout C, Agudelo CA, et al (2012) Histoplasmosis: results of the Colombian national survey, 1992–2008. Biomedica 31: 344–356. 10.1590/S0120-41572011000300007 22674311

[4] Bava AJ (1995) Histoplasmosis in the Muniz Hospital of Buenos Aires. Rev Inst Med Trop Sao Paulo 37: 531–535. 8731267 10.1590/s0036-46651995000600010

[5] Brilhante RS, Fechine MA, Mesquita JR, Cordeiro RA, Rocha MF, et al (2012) Histoplasmosis in HIV-positive patients in Ceara, Brazil: clinical-laboratory aspects and in vitro antifungal susceptibility of Histoplasma capsulatum isolates. Trans R Soc Trop Med Hyg 106: 484–488. 22703696 10.1016/j.trstmh.2012.05.003

[6] Cermeno JR, Hernandez I, Godoy G, Cabello I, Cermeno JJ, et al (2005) [Mycoses at Hospital Universitario “Ruiz y Paez,” Ciudad Bolivar, Venezuela, 2002]. Invest Clin 46: 37–42. 15782535

[7] Daher EF, Silva GB Jr, Barros FA, Takeda CF, Mota RM, et al (2007) Clinical and laboratory features of disseminated histoplasmosis in HIV patients from Brazil. Trop Med Int Health 12: 1108–1115. 17875020 10.1111/j.1365-3156.2007.01894.x

[8] de Francesco Daher E, de Sousa Barros FA, da Silva Junior GB, Takeda CF, Mota RM, et al (2006) Risk factors for death in acquired immunodeficiency syndrome-associated disseminated histoplasmosis. Am J Trop Med Hyg 74: 600–603. 16606992

[9] Eza D, Cerrillo G, Moore DA, Castro C, Ticona E, et al (2006) Postmortem findings and opportunistic infections in HIV-positive patients from a public hospital in Peru. Pathol Res Pract 202: 767–775. 16979302 10.1016/j.prp.2006.07.005 PMC2912516

[10] Guimaraes LC, Silva AC, Micheletti AM, Moura EN, Silva-Vergara ML, et al (2012) Morphological changes in the digestive system of 93 human immunodeficiency virus positive patients: an autopsy study. Rev Inst Med Trop Sao Paulo 54: 89–93. 22499422 10.1590/s0036-46652012000200006

[11] Mora DJ, dos Santos CT, Silva-Vergara ML (2008) Disseminated histoplasmosis in acquired immunodeficiency syndrome patients in Uberaba, MG, Brazil. Mycoses 51: 136–140. 18254750 10.1111/j.1439-0507.2007.01459.x

[12] Pietrobon D, Negro-Marquinez L, Kilstein J, Galindez J, Greca A, et al (2004) Disseminated histoplasmosis and AIDS in an Argentine hospital: clinical manifestations, diagnosis and treatment. Enferm Infecc Microbiol Clin 22: 156–159. 14987536 10.1016/s0213-005x(04)73056-6

[13] Pontes LB, Leitao Tdo M, Lima GG, Gerhard ES, Fernandes TA (2010) Clinical and evolutionary characteristics of 134 patients with disseminated histoplasmosis associated with AIDS in the State of Ceara. Rev Soc Bras Med Trop 43: 27–31. 20305964 10.1590/s0037-86822010000100007

[14] Davel G, Canteros CE (2007) Epidemiological status of mycoses in the Argentine Republic. Rev Argent Microbiol 39: 28–33. 17585656

[15] Tobon AM, Agudelo CA, Rosero DS, Ochoa JE, De Bedout C, et al (2005) Disseminated histoplasmosis: a comparative study between patients with acquired immunodeficiency syndrome and non-human immunodeficiency virus-infected individuals. Am J Trop Med Hyg 73: 576–582. 16172484

[16] Tobón AMMA, Orozco L, Restrepo C, Molina D, de Bedout C (2011) Histoplasmosis diseminada en una cohorte de pacientes coinfectados con el VIH. Acta Med Colombiana. Acta Med Colombiana 36: 63–67.

[17] Peigne V, Dromer F, Elie C, Lidove O, Lortholary O (2011) Imported acquired immunodeficiency syndrome-related histoplasmosis in metropolitan France: a comparison of pre-highly active anti-retroviral therapy and highly active anti-retroviral therapy eras. Am J Trop Med Hyg 85: 934–941. 22049053 10.4269/ajtmh.2011.11-0224 PMC3205645

[18] Antinori S, Magni C, Nebuloni M, Parravicini C, Corbellino M, et al (2006) Histoplasmosis among human immunodeficiency virus-infected people in Europe: report of 4 cases and review of the literature. Medicine (Baltimore) 85: 22–36. 16523050 10.1097/01.md.0000199934.38120.d4

[19] Buitrago MJ, Gomez-Lopez A, Monzon A, Rodriguez-Tudela JL, Cuenca-Estrella M (2007) [Assessment of a quantitative PCR method for clinical diagnosis of imported histoplasmosis]. Enferm Infecc Microbiol Clin 25: 16–22. 17261242 10.1157/13096748

[20] Couppie P, Aznar C, Carme B, Nacher M (2006) American histoplasmosis in developing countries with a special focus on patients with HIV: diagnosis, treatment, and prognosis. Curr Opin Infect Dis 19: 443–449. 16940867 10.1097/01.qco.0000244049.15888.b9

[21] Huber F, Nacher M, Aznar C, Pierre-Demar M, El Guedj M, et al (2008) AIDS-related Histoplasma capsulatum var. capsulatum infection: 25 years experience of French Guiana. AIDS 22: 1047–1053. 18520348 10.1097/QAD.0b013e3282ffde67

[22] Assi MA, Sandid MS, Baddour LM, Roberts GD, Walker RC (2007) Systemic histoplasmosis: a 15-year retrospective institutional review of 111 patients. Medicine (Baltimore) 86: 162–169. 17505255 10.1097/md.0b013e3180679130

[23] Gutierrez ME, Canton A, Sosa N, Puga E, Talavera L (2005) Disseminated histoplasmosis in patients with AIDS in Panama: a review of 104 cases. Clin Infect Dis 40: 1199–1202. 15791523 10.1086/428842

[24] Bulmer AC, Bulmer GS (2001) Incidence of histoplasmin hypersensitivity in the Philippines. Mycopathologia 149: 69–71. 11265164 10.1023/a:1007277602576

